# Commentary: Is the Frontal Lobe Involved in Conscious Perception?

**DOI:** 10.3389/fpsyg.2015.01736

**Published:** 2015-11-12

**Authors:** Marnix Naber, Jan Brascamp

**Affiliations:** ^1^Department of Experimental Psychology, Helmholtz Institute, Utrecht UniversityUtrecht, Netherlands; ^2^Department of Psychology, Michigan State University, East LansingMI, USA

**Keywords:** prefrontal cortex, frontal lobe, conscious awareness, perceptual switching, content of consciousness, conscious perception, binocular rivalry, visual perception

Like any other field, the field of consciousness research benefits from a careful distinction between the concepts involved. An example is the distinction between the state of being conscious (e.g., whether someone is awake) and the contents of consciousness (e.g., whether someone perceives a dress as white or blue). A similar type of distinction can contribute to the resolution of a debate regarding the role of the frontal cortex in conscious perception. In a recent publication Safavi et al. (2014) responded to conclusions drawn in a study regarding the role of frontal cortex in conscious perception. This study, by Frässle et al. (2014), employed binocular rivalry to show that well-established frontal BOLD correlates of perceptual switches (Lumer et al., 1998; Sterzer and Kleinschmidt, 2007) were strongly diminished when participants passively viewed these switches rather than reporting them. Frässle and co-workers (among whom the present paper's first author) concluded: “*frontal areas are associated with active report and introspection rather than with rivalry per se*.” This statement is a bit audacious, as it rules out any role of frontal areas in rivalry other than their role in reporting perception. As such, Safavi and co-workers draw into question this conclusion, based on evidence indicating that neural activity in frontal areas, in particular in the lateral prefrontal cortex, reflects the contents of consciousness in paradigms that do not involve active report.

Here we aim to bring these two positions closer together by making an explicit distinction between neural activity that reflects the *content* of consciousness, and neural activity that brings about *changes* in consciousness during perceptual bistability. Safavi et al. (2014) bring forward a variety of studies linking frontal lobe function to the content of consciousness (Imamoglu et al., 2012; Panagiotaropoulos et al., 2012). Frässle and colleagues, in turn, are specifically concerned with fMRI BOLD responses that are time-locked to perceptual switches, not to periods when the contents of consciousness are stable. Recent debate has focused on the question what brings about this switch-related BOLD signal during bi-stable perception, with a central question being whether it reflects a neural initiation of these switches or an indirect consequence (Sterzer et al., 2009; Knapen et al., 2011; Weilnhammer et al., 2013; Frässle et al., 2014). Frässle and colleagues' conclusions should be understood in the context of this question. Specifically, the conclusions promote the idea that the frontal response observed during binocular rivalry reflects an indirect consequence of perceptual switches, as it is associated with reporting them. In other words, while we do not disagree with Safavi et al.'s arguments regarding a potential role of frontal cortex in representing the contents of conscious perception, and while we recognize that Frässle and colleagues perhaps have oversimplified the dichotomy between report-related and initiation-related functions, these concerns should not obscure Frässle et al.'s point with regard to frontal cortex' involvement in perceptual switches during bi-stable perception.

As a further point, the “binocular flash suppression” paradigm that provides much of the empirical basis of Safavi and colleagues' argument, manipulates the content of awareness while explicitly avoiding spontaneous switches in perception. This provides a further indication that Frässle et al.'s claims regarding perceptual switches can exist side-by-side with Safavi et al.'s claims regarding stable perception. Indeed, the next paragraph will summarize evidence that frontal involvement in coding the contents of consciousness does not imply frontal involvement in perceptual switches.

Whereas the anatomical distribution of activity that reflects the content of consciousness during bi-stable perception depends on the stimulus employed (Logothetis and Schall, 1989; Leopold and Logothetis, 1996; Tong et al., 1998; Andrews et al., 2002; Parker et al., 2002), the locus of fMRI BOLD concomitants of perceptual switches is remarkably constant across distinct forms of perceptual bistability (Sterzer and Kleinschmidt, 2007; Knapen et al., 2011; Frässle et al., 2014). Evidence from studies on the peripheral nervous system with pupillometry points to a similar distinction between mechanisms involved in stable vs. instable perception. The eye's pupil can track the perceptual content of consciousness when this content changes in terms of luminance or contrast: the pupil constricts when a bright, high-contrast percept is experienced and dilates when a dark, low-contrast percept is experienced (Naber et al., 2011). This directionality is unrelated to a different pupillary response: a brief pupil dilation around the time of a perceptual switch, independent of luminance or contrast change (Einhäuser et al., 2008; Hupé et al., 2009; Naber et al., 2011). The phenomenological difference between these pupil responses reflects distinct neural origins: the pupil's reflection of perceptual content (i.e., contrast/luminance) is driven by higher-level cortical visual areas (Barbur et al., 1992, 1998; Naber and Nakayama, 2013) while pupil responses during perceptual switches are the result of sympathetic arousal (Bradshaw, 1967; Einhäuser et al., 2008; Preuschoff et al., 2011; Laeng et al., 2012; Stoll et al., 2013). Pupillary dynamics during rivalry hence underscore the distinction between processes that reflect the content of consciousness and processes involved in changes in this content.

As a final remark we would like to stress, as others have (Safavi et al., 2014; Zaretskaya and Narinyan, 2014) that several frontal areas still showed switch-related BOLD activity in Frässle et al.'s condition without active report. In particular, the right superior frontal gyrus (RSFG) and right inferior frontal gyrus (RIFG) remained active without the report, suggesting that these areas may still be causally involved in perceptual transitions (Sterzer and Kleinschmidt, 2007; Weilnhammer et al., 2013). This possibility, however, remains disputed at this point (Knapen et al., 2011; Weilnhammer et al., 2013; Brascamp et al., 2015), and a recent study pinpoints the RIFG as a salient event detector rather than an executive controller (Hampshire et al., 2010).

In conclusion, we agree with the notion, emphasized by Safavi and colleagues, that the field of consciousness research will benefit from an integrated view of evidence from various experimental and neuroscientific paradigms (Aru and Bachmann, 2015). Future research on consciousness will also benefit from careful distinction of the exact roles of different frontal lobe areas (Zaretskaya and Narinyan, 2014). Here we add to that the importance of a careful distinction between various processes and mechanisms that together contribute to conscious experience.

## Conflict of interest statement

The authors declare that the research was conducted in the absence of any commercial or financial relationships that could be construed as a potential conflict of interest.
